# Advancing HIV Diagnostics: Comparative Evaluation of Multisure HIV-1/2 Rapid Confirmatory Test Against Geenius and Traditional Reference Assays Within a CDC-Aligned Diagnostic Framework

**DOI:** 10.3390/microorganisms14030693

**Published:** 2026-03-19

**Authors:** Ahmed Ismail, Israa M. Salameh, Nadin Younes, Parveen B. Nizamuddin, Shaden Abunasser, Salma Younes, Sara Abdelmohsen, Mazen N. Abouassali, Manal Elshaikh, Ibrahim W. Karimeh, Mohammed A. Ibrahim, Mutaz M. Ali, Ibrahim Al Shaar, Haris Ong, Çiğdem S. Zhmurov, Hadi M. Yassine, Laith J. Abu-Raddad, Houssein Ayoub, Gheyath K. Nasrallah

**Affiliations:** 1 Laboratory Section, Medical Commission Department, Ministry of Public Health, Doha P.O. Box 42, Qatar; aismail@moph.gov.qa (A.I.); mnajib@moph.gov.qa (M.N.A.); mibrahim1@moph.gov.qa (M.E.); ikarime@moph.gov.qa (I.W.K.); mabdulfatah@moph.gov.qa (M.A.I.); mmohamoud@moph.gov.qa (M.M.A.); ialshaar@moph.gov.qa (I.A.S.); 2 Biomedical Research Center, Qatar University, Doha 2713, Qatar; israa.salameh46@gmail.com (I.M.S.); ny1204022@student.qu.edu.qa (N.Y.); parveen.n@qu.edu.qa (P.B.N.); sa1708343@student.qu.edu.qa (S.A.); sy1203986@student.qu.edu.qa (S.Y.); hyassine@qu.edu.qa (H.M.Y.); 3 Department of Biomedical Science, College of Health Sciences, Qatar University Health Sector, Qatar University, Doha 2713, Qatar; sz2105828@student.qu.edu.qa; 4 MP Biomedicals Asia Pacific Pte. Ltd., 2 Pioneer Place, Singapore 627885, Singapore; haris.ong@mpbio.com; 5 Department of Molecular Biology and Genetics, Graduate School of Science, Üsküdar University, Istanbul 34662, Türkiye; cigdem.sezerzhmurov@uskudar.edu.tr; 6 Infectious Disease Epidemiology Group, Weill Cornell Medicine-Qatar, Cornell University, Qatar Foundation-Education City, Doha 24144, Qatar; lja2002@qatar-med.cornell.edu; 7 World Health Organization Collaborating Centre for Disease Epidemiology Analytics on HIV/AIDS, Sexually Transmitted Infections, and Viral Hepatitis, Weill Cornell Medicine-Qatar, Cornell University, Qatar Foundation-Education City, Doha 24144, Qatar; 8 Department of Healthcare Policy and Research, Weill Cornell Medicine, Cornell University, New York, NY 10065, USA; 9 Mathematics Program, Department of Mathematics and Statistics, College of Arts and Sciences, Qatar University, Doha 2713, Qatar; hayoub@qu.edu.qa

**Keywords:** rapid diagnostic tests, HIV confirmatory assays, HIV-1/2 differentiation, HIV diagnostic algorithm

## Abstract

Human immunodeficiency virus (HIV) remains a major global health challenge, requiring accurate diagnostic testing for early detection. Chemiluminescent immunoassay screening, particularly the Architect HIV Ag/Ab Combo assay, followed by immunoblot confirmation using INNO-LIA™ has traditionally been used in many diagnostic workflows. To address these limitations, the U.S. Centers for Disease Control and Prevention (CDC) recommends the use of an HIV-1/2 antibody differentiation immunoassay, such as the Geenius HIV-1/2 Supplemental Assay, as part of the confirmatory testing algorithm. This study evaluates the performance of two rapid HIV-1/2 confirmatory assays—the Multisure HIV-1/2 Confirmatory Test and the Bio-Rad Geenius HIV-1/2 Supplemental Assay—within a CDC-aligned diagnostic framework, with the aim of assessing Multisure as a potential alternative differentiation assay. A total of 224 archived serum samples were analyzed, including true positives (*n* = 38), true negatives (*n* = 139), false positives (*n* = 20), and INNO-LIA™ indeterminate samples (*n* = 27), as defined by Architect HIV and INNO-LIA™ results. Samples were initially screened using the Architect HIV Ag/Ab Combo assay, confirmed by INNO-LIA™ and PCR, and subsequently re-tested using Multisure HIV-1/2 and Geenius HIV-1/2 assays. Diagnostic performance metrics were evaluated. Both rapid assays demonstrated 100% sensitivity and specificity when compared with INNO-LIA™. Among INNO-LIA™ indeterminate samples, Multisure HIV-1/2 classified 81.5% as negative compared with 55.6% using Geenius HIV-1/2. When compared with PCR, Multisure demonstrated higher specificity (89.2%) and positive predictive value (89.5%) than Geenius (82.9% and 84.6%). No confirmed HIV-2 infections were identified in the analyzed dataset, and HIV-1 subtype information was not available for the archived samples; therefore, conclusions regarding HIV-1/2 differentiation are based primarily on assay design and antigenic targets. Multisure HIV-1/2 demonstrated strong diagnostic performance comparable to established differentiation assays and may represent a practical alternative rapid confirmatory option within CDC-aligned HIV diagnostic workflows. Further studies including larger datasets and confirmed HIV-2 infections are warranted to further validate its clinical utility.

## 1. Introduction

The human immunodeficiency virus (HIV), of the family *Retroviridae* and genus *Lentivirus*, remains a major global public health threat and is one of the primary infections transmitted through blood transfusions [1]. In 2023, 1.3 million new HIV infections were reported, with 39.9 million people living with the virus and 630,000 AIDS-related deaths [2]. The rapid spread of HIV underscores the urgent need for reliable and accessible diagnostic methods as part of heightened prevention strategies [3,4]. Addressing this need enables early detection and treatment, thereby preventing adverse individual and public health outcomes [5,6,7].

Third-generation enzyme-linked immunosorbent assays (ELISAs) were historically used as primary screening tools [8]. Although still valuable, ELISA has been largely replaced by more sensitive fourth-generation assays such as the Architect HIV Ag/Ab Combo [8,9,10]. In Qatar, particularly at the Medical Commission—the setting of this study—the Architect HIV Ag/Ab Combo assay serves as the routine fourth-generation screening method.

HIV testing strategies vary globally, including routine, integrated, and community-based testing [11]. In Qatar, routine HIV testing is the primary detection strategy [12]. In 2014, the CDC and the Association of Public Health Laboratories (APHL) published an updated laboratory diagnostic algorithm for HIV infection, recommending the use of an FDA-approved fourth-generation HIV-1/2 antigen/antibody immunoassay for initial screening, followed by an FDA-approved HIV-1/HIV-2 antibody differentiation immunoassay for confirmatory testing. If the differentiation assay yields non-reactive or indeterminate results, an HIV-1 nucleic acid test (NAT) is recommended to identify acute infection. This algorithm and its reporting framework were further clarified in a 2018 technical update. At the time of publication, the Geenius HIV-1/2 Supplemental Assay was the only FDA-approved differentiation immunoassay referenced in the CDC reporting guidance [13,14]. At the Medical Commission (MC), applicants immigrating to the country undergo routine HIV screening, initially performed using the ARCHITECT^®^ HIV Ag/Ab Combo assay (Abbott Diagnostics, Abbott Park, IL, USA). Samples that show reactivity to HIV antigen and/or antibodies are retested in duplicate. If both replicates are non-reactive, the sample is reported as non-reactive. If one or both replicates are reactive, the sample is considered reactive.

All initially reactive samples from the first HIV Ag/Ab Combo test (Run 1, Analyzer A) are subsequently retested using a second ARCHITECT^®^ HIV Ag/Ab Combo assay (Analyzer B) and two supplementary tests, INNO-LIA™ HIV I/II Score and RT-PCR, performed on freshly drawn blood samples (Run B) [9,12].

Despite advancements in screening methods, some laboratories continue to rely on line immunoassays (LIA), such as INNO-LIA™, as supplemental confirmatory tests, which has shown an inability to classify a significant proportion of samples as definitive positive or negative, resulting in indeterminate (IND) results [15,16,17]. An IND result indicates a non-definitive outcome, where antibodies are detected but a conclusive outcome cannot be specified as positive or negative. Furthermore, INNO-LIA™ demonstrates reduced sensitivity to HIV-2, necessitating further testing [18].

The development of rapid antibody differentiation assays, such as the Geenius HIV-1/2 Supplemental Assay, has improved HIV confirmatory testing by providing faster and more standardized differentiation between HIV-1 and HIV-2 infections [19]. Recent studies have focused on investigating such confirmatory RDTs as a replacement for both screening and confirmatory assays [20,21]. Several studies have demonstrated that rapid tests outperform immunoblots in key diagnostic aspects [20], specifically when it comes to differentiating between HIV-1 and HIV-2 instances [15,18,20,22]. This distinction is clinically important as HIV-2 is less responsive to certain antiretroviral drugs [23]. Although HIV-2 infections are relatively rare in many screening populations, including Qatar, confirmatory assays capable of differentiating HIV-1 from HIV-2 remain essential for appropriate clinical management [18].

The growing demand for confirmatory RDTs reflects the need for affordable and accessible diagnostic tools, especially in resource-limited settings [24,25,26]. Immunoblot-based assays can cost up to three times more than rapid tests [24], making them impractical for widespread use in low-resource regions [27]. Rapid confirmatory tests provide same-day results and can be deployed in community-based settings, expanding access to diagnostics in limited healthcare infrastructure [28]. This accessibility plays a critical role in preventing the spread of HIV by ensuring that undiagnosed individuals are identified and linked to care quickly [28].

This study evaluates and compares the performance of rapid HIV-1/2 antibody differentiation assays—the CDC-recommended Geenius HIV 1/2 Supplemental Assay (Bio-Rad Laboratorie, CA, USA) and the Multisure HIV 1/2 Confirmatory Test (MP Biomedicals Asia Pacific Pte Ltd., Pioneer Place, Singapore)—against the reference INNO-LIA™ assay (Innogenetics, Ghent, Belgium; now: Fujirebio Europe N.V.). It seeks to demonstrate their concordance with INNO-LIA™ and the potential of incorporating the Multisure HIV1/2 confirmatory rapid test into the CDC HIV diagnostic algorithm. Additionally, this study investigates their ability to resolve the indeterminate (IND) results often seen with INNO-LIA™, illustrating more definitive outcomes. While Geenius HIV1/2 has been previously studied [18,29,30,31], the performance of the Multisure HIV1/2 test remains underexplored, representing a novel contribution of this study to the literature [32].

## 2. Materials and Methods

### 2.1. Ethical Approval

Previously collected serum samples from our previous studies were utilized [9,12]. This study received approval from the Qatar University Institutional Review Board (QU-IRB 017/2024-E).

### 2.2. Study Population and Study Design

This retrospective diagnostic accuracy study was conducted using archived samples following the MC testing protocol. Demographic data (sex and race/ethnicity) and collection dates were unavailable, as anonymized specimens were used.

A total of 224 samples were selected, providing sufficient statistical power for comparative performance assessment. The MC operates under the Ministry of Public Health (MOPH). As part of Qatar’s recruitment procedures, the MC performs several medical tests, including routine HIV1/2 screening of applicants. MC re-tests discrepant (indeterminate) samples four weeks post-initial testing with fresh blood for confirmation. In this study, archived HIV1/2 samples were provided by the MC under a test-result-dependent coding system that ensured confidentiality. The coding system was based on Architect HIV Ag/Ab combo screening and INNO-LIA™ results. There was no patient recruitment, nor was there any direct or indirect contact with any of the study’s subjects.

Out of the large dataset provided, samples showing both concordance and discordance between Architect HIV and INNO-LIA™ were selected for comprehensive comparison. The dataset included true positives (*n* = 38; Architect HIV positive & INNO-LIA™ positive, PP), true negatives (*n* = 139; Architect HIV negative & INNO-LIA™ negative, N), false positives (*n* = 20; Architect HIV positive & INNO-LIA™ negative, PN), and indeterminate results (*n* = 27). The indeterminate group comprised 22 PI samples (Architect HIV positive & INNO-LIA™ indeterminate) and 5 samples negative by Architect HIV but indeterminate by INNO-LIA™.

**Figure 1:**
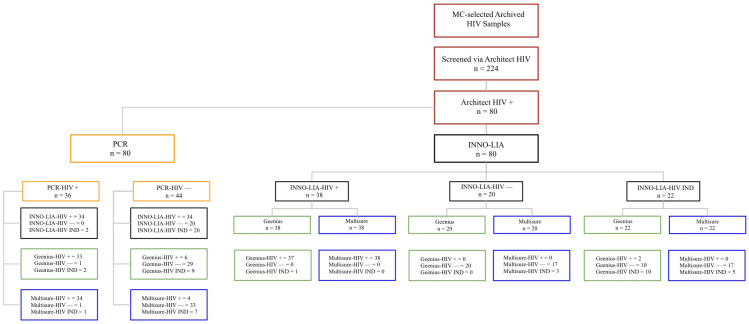
Flowchart outlining the analysis of Architect-HIV-positive samples (*n* = 80). All positive samples were tested by PCR, INNO-LIA™, Geenius HIV1/2, and Multisure HIV1/2 as part of the confirmatory workflow.

**Figure 2:**
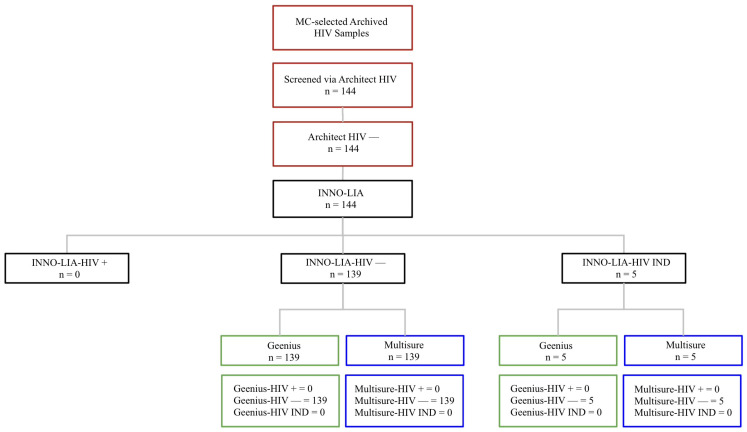
Flowchart outlining the analysis of Architect-HIV-negative samples (*n* = 144). These samples were re-tested by INNO-LIA™, Geenius HIV1/2, and Multisure HIV1/2, while PCR was not performed according to MC standard procedure.

In total, 224 samples were initially screened using the Architect HIV Ag/Ab combo assay. Confirmatory testing was performed with INNO-LIA™, which served as the gold standard reference, and PCR was conducted only for Architect-positive samples. All samples were subsequently evaluated with Multisure HIV1/2 and Geenius HIV1/2, as outlined in Figure 1 (Architect-positive workflow) and Figure 2 (Architect-negative workflow).

### 2.3. Abbott Architect HIV Ag/Ab Combo Assay

The Architect HIV Ag/Ab Combo Assay (Abbott Diagnostics, Abbott Park, IL, USA) is a chemiluminescent test that detects both HIV p24 antigen and antibodies to HIV-1 (groups M and O) and HIV-2 in human serum and plasma (EDTA and heparin) [33].

According to MC laboratory protocols, any reactive sample undergoes re-testing. If both tests return non-reactive, the sample is considered non-reactive. However, if one or both tests are reactive, the sample is confirmed as reactive. All samples initially reactive from the Architect HIV assay were re-tested using Architect HIV analyser, INNO-LIA™, and PCR using fresh blood.

### 2.4. Fujirebio INNO-LIA™ HIV I/II Score

The INNO-LIA™ is a line immuno-assay (Innogenetics, Ghent, Belgium; now: Fujirebio Europe N.V.) used to confirm antibodies against HIV-1 (including group O) and HIV-2 in human serum or plasma as described previously [9,12,34]. It is able to differentiate between HIV-1 and HIV-2 infections [34]. Test strips coated with recombinant proteins and synthetic HIV1/2 peptides are present. All strips tested were examined and interpreted using automated LiRAS for Infectious Diseases software v4.00 CE [34], specifically designed for the interpretation of LIA results, all in accordance with the manufacture instruction of the kit.

### 2.5. Bio-Rad Geenius HIV1/2 Supplemental Assay

The Geenius HIV1/2 Supplemental Assay (Hercules, CA, US) is a lateral flow test that uses a cassette with HIV-1 and HIV-2 recombinant antigens attached to a membrane. As the sample migrates along the test strip, specific anti-HIV antibodies are captured by immobilized recombinant antigens representing HIV-1 and HIV-2. Visible bands corresponding to HIV1/2 antibodies appear if present [35].

### 2.6. MP Diagnostics Multisure HIV1/2 Confirmatory Test

The Multisure HIV1/2 Confirmatory Test (MP Biomedicals Asia Pacific, Singapore) is a rapid qualitative test for detecting and differentiating antibodies of HIV-1 and HIV-2 in human serum or plasma. It features highly purified recombinant antigens (gp120, gp41, and p24 for HIV-1 and gp36 and gp105 for HIV-2) arranged in five test lines on a membrane. As the sample migrates upward from the sample well, antibodies form complexes with the immobilized antigens [36]. Bands to corresponding antibodies appear if present. Band interpretation was conducted using the iPeak 4.3” Lateral Flow Reader as per manufacturer guidelines [36].

### 2.7. Statistical Analysis

The collected dataset was comprised of categorical data; as a result, descriptive statistical analysis was performed. To successfully assess concordance of Multisure HIV1/2 and Geenius HIV1/2 against standard references, performance evaluation metrics were measured as previously described [37,38]. These metrics included Sensitivity, Specificity, Positive Predictive Value (PPV), Negative Predictive Value (NPV), Overall Percent Agreement (OPA), Positive Percent Agreement (PPA), Negative Percent Agreement (NPA) and agreement coefficient, Cohen’s Kappa. Level of agreement values of 95% confidence interval (CI) are considered as follows: *κ* < 0 indicates no agreement, *κ* = 0.00–0.20 indicates slight agreement, *κ* = 0.21–0.40 indicates fair agreement, *κ* = 0.41–0.60 indicates moderate agreement, *κ* = 0.61–0.80 indicates substantial agreement and *κ* = 0.81–1.00 indicates almost perfect agreement [39,40]. All statistical analyses were conducted using GraphPad Prism software, version 10.4.1 (San Diego, CA, US).

## 3. Results

### 3.1. Multisure HIV1/2 and Geenius HIV1/2 Demonstrate Excellent Sensitivity and Specificity with No False Positives or False Negatives Compared to INNO-LIA™ Confirmatory Assay

A total of 224 samples tested with INNO-LIA™ were used as the basis for assessing the performance of Multisure HIV1/2 and Geenius HIV1/2. Out of this sample set, 27 IND samples were excluded from statistical analysis. Multisure HIV1/2 identified 17% (38/224) of the samples as true positive and 69.6% (156/224) as true negative. Notably, no false positives or false negatives were observed when comparing Multisure HIV1/2 to INNO-LIA™. Indeterminate results accounted for 3.5% (8/224) of the cases.

Similarly, when assessing Geenius HIV1/2, there were no false positives nor false negatives. It was found that 16.5% (37/224) were confirmed as true positives and 71% (159/224) were confirmed as true negatives, while 5% (11/224) of cases presented as IND (Table 1). It is of importance to highlight that rapid confirmatory testing was performed once per sample according to the manufacturer’s instructions using archived specimens; repeat testing of indeterminate rapid test results was not performed due to sample availability constraints. Therefore, reproducibility of indeterminate results could not be assessed in this study.

Performance evaluation metrics were applied to assess the concordance of Multisure HIV1/2 and Geenius HIV1/2 relative to INNO-LIA™. Multisure HIV1/2 and Geenius HIV1/2 demonstrated 100% specificity and sensitivity in the detection of HIV1/2, indicating that both are able to recognize negative and positive cases accurately. This concordance is further supported via the 100% OPA, PPA, NPA, PPV and NPV, exhibiting the kits’ ability to successfully reflect the state of infection. Similarly, Cohen’s Kappa coefficient (*κ* = 1.000) confirmed perfect agreement between INNO-LIA™ and both rapid tests (Table 2). Using Fisher’s Exact test, these findings were deemed statistically significant (*p* < 0.001).

### 3.2. Multisure Demonstrated Slightly Higher Specificity and Fewer Indeterminate Results in This Dataset

A total of 80 PCR samples were utilized to assess Multisure HIV1/2 and Geenius HIV1/2. In total, 8 and 11 IND samples were excluded during statistical analysis in Multisure HIV1/2 and Geenius HIV1/2, respectively. When evaluating Multisure HIV1/2, it demonstrated 42.5% (34/80) as true positives and 41.3% (33/80) as true negatives. The rapid test recorded a single false negative (1.3%) and 5% (4/80) as false positives. Using Fisher’s Exact test, these findings were deemed statistically significant (*p* < 0.001).

A total of 10.1% (8/80) of the cases were defined as IND, where 2.8% PCR-positive samples (1/36) and 15.9% PCR-negative samples (7/44) were classified as indeterminate by Multisure HIV1/2 (Table 1). Likewise, when evaluating Geenius HIV1/2, 41.3% (33/80) presented as true positives and 36.3% (29/80) as true negatives. Similar to Multisure HIV1/2, Geenius HIV1/2 also presented a single false negative (1.3%). A total of 7.5% (6/80) of the cases were found to be false positives. Total IND samples made up 13.8% (11/80), where 5.6% PCR-positive samples (2/36) and 20.5% PCR-negative samples (9/44) were classified as indeterminate by Geenius HIV1/2 (Table 1).

In light of these findings, performance evaluation metrics were considered. Both Multisure HIV1/2 and Geenius HIV1/2 showed high sensitivity at 97.1%. The specificity for Multisure HIV1/2 outperformed that of Geenius HIV1/2, where it was found to be 89.2% and 82.9%, respectively. This was also true regarding PPV, NPV and OPA, where Multisure HIV1/2 recorded 89.5%, 97.1% and 93.1%, respectively. On the other hand, Geenius HIV1/2 recorded 84.6% PPV, 96.7% NPV and 89.9% OPA. PPA for both tests were 97.1% and NPA was observed as 89.2% for Multisure HIV1/2 and 82.9% for Geenius HIV1/2. Moreover, Cohen’s Kappa was considered, where Multisure HIV1/2 was 0.861 and Geenius HIV1/2 was 0.797. Using Fisher’s Exact test, these findings were deemed statistically significant (*p* < 0.001). Overall, the analysis shows that both rapid tests are able to identify individuals who do not have the disease and those who do to a high caliber. Additionally, it can be concluded from Cohen’s Kappa that Multisure HIV1/2 had almost perfect agreement, while Geenius had substantial agreement. Overall, while these indeterminate outcomes introduce a degree of clinical uncertainty, both tests maintained strong diagnostic performance, particularly Multisure HIV1/2, which showed fewer discrepancies and indeterminate results across both positive and negative PCR cases.

## 4. Discussion

This study found that both Multisure HIV1/2 and Geenius HIV1/2 demonstrated excellent performance when compared to INNO-LIA™ and showed high overall concordance with PCR. While both rapid tests showed perfect results with INNO-LIA™, analysis against PCR showed slight discrepancies. It is important to note that IND results were excluded from the performance evaluations of both rapid tests against INNO-LIA™ and PCR, as is standard practice in such analyses; IND cases were analyzed separately.

In the performance evaluation of Multisure HIV1/2 and Geenius HIV1/2 against PCR, due to the presence of a single false negative in both Multisure HIV1/2 and Geenius HIV1/2, the tests shared identical and nearly perfect sensitivity and NPV (97.1%). As for false positive results, Multisure HIV1/2 had fewer instances recorded than Geenius HIV1/2 (in a 2:3 ratio); nonetheless, it influenced its performance. Specifically, Multisure HIV1/2 achieved very good specificity and PPV (89.2% and 89.5%). As for Geenius HIV1/2, good specificity and PPV (82.9% and 84.6%) and very good OPA (89.9%) were observed. The PPV values observed in this study should be interpreted in the context of the study design. The sample panel intentionally included a high proportion of discordant and indeterminate cases to evaluate the diagnostic performance of rapid confirmatory assays in challenging scenarios. Such enrichment increases the probability of borderline serological patterns and therefore may reduce PPV estimates compared with routine screening populations. In real-world diagnostic workflows with lower proportions of discordant samples, higher PPV values would be expected. Substantial agreement was also seen (*κ* = 0.797). Despite discrepancies, Multisure HIV1/2 showed slightly higher specificity and PPV than Geenius HIV1/2. This is evident from the higher false positives in Geenius HIV1/2 (6/80). Although PCR is a highly sensitive molecular method, discordance between molecular and serological assays may reflect differences in biological targets and timing of infection rather than analytical failure of a specific platform. PCR was used in this study as an additional molecular reference to further evaluate discordant cases rather than as a definitive gold standard for infection status. Molecular assays detect viral RNA, whereas serological assays detect host antibody responses, and discrepancies may reflect differences in biological targets or stage of infection. Because the study relied on anonymized archived samples without longitudinal follow-up, the true infection status of discordant cases could not be independently adjudicated. Therefore, discordant findings should be interpreted cautiously within the diagnostic framework rather than attributed to a specific platform [41]. This highlights the need for caution when interpreting discordant findings and emphasizes the importance of using multiple diagnostic tools in parallel to ensure accurate HIV detection. Despite these discrepancies, both Multisure HIV1/2 and Geenius HIV1/2 demonstrated high overall percent agreement (OPA) and exhibited performance consistent with their role as robust alternatives to traditional immunoblot assays. The results suggest that rapid confirmatory tests could provide reliable diagnostic outcomes, even in cases where confirmatory PCR results appear inconsistent.

In addition to the primary performance evaluation, a secondary analysis was conducted focusing on samples that initially yielded IND results by INNO-LIA™. Although excluded from sensitivity and specificity calculations, as per standard evaluation protocols, the discrepancies observed in this subset provided valuable insights into the comparative strengths and limitations of Multisure HIV1/2 and Geenius HIV1/2.

For INNO-LIA™ IND cases (*n* = 27), 81.5% (22/27) of samples tested via Multisure HIV1/2 presented as negative, whereas only 5/27 samples presented as IND, highlighting its ability to resolve 22/27 of the cases as definitive negative. On the other hand, Geenius HIV1/2 identified 37% of the cases as IND. The remaining 63% mainly consisted of negative samples (15/27), with the exception of two positive cases. When compared to PCR and Multisure HIV1/2, these two particular samples were seen as negative. Discordance between molecular and serological assays may reflect differences in biological targets and timing of infection rather than analytical error of a specific platform. Molecular assays detect viral RNA, whereas serological assays detect host antibody responses, and discrepancies may occur during transitional stages of infection [42]. Indeterminate results in HIV confirmatory assays are well documented and may arise from multiple biological and technical factors. These include early seroconversion prior to complete antibody maturation, waning antibody responses in advanced infection, cross-reactive antibodies, autoimmune disorders, pregnancy, and coinfections, as well as nonspecific reactivity related to specimen quality. Because both immunoblots and rapid differentiation assays detect host antibody responses rather than viral nucleic acid, indeterminate patterns may reflect transitional serological stages rather than analytical failure of the assay. Similar mechanisms have been described previously in studies investigating discordant and indeterminate HIV serology patterns [41,42,43].

Collectively, it is concluded that both Multisure HIV1/2 and Geenius HIV1/2 are able to resolve IND cases present in INNO-LIA™ [9,12,18,20,43,44]. This has been previously demonstrated, where even rapid HIV tests utilized for screening were able to conclude definitive results when immunoblot assays failed to do so [42]. Specifically, several studies have observed this in Geenius HIV1/2 as well [15,18,45]. Multisure HIV1/2, however, has not been studied in this context previously, and so is a novel finding in this study. It is worth mentioning that Multisure HIV1/2 resolved IND cases more effectively than Geenius, classifying them mainly as negative, supporting its reliability.

When analyzing INNO-LIA™ negative samples, three cases were identified by Multisure HIV1/2 as IND, while Geenius HIV1/2 categorized them as negative (Table 1). This highlights an important distinction between the two rapid tests. While Multisure HIV1/2 demonstrated greater efficacy in resolving INNO-LIA™ IND cases, Geenius HIV1/2 exhibited a higher sensitivity in accurately categorizing true negative samples. This may suggest a slight advantage for Geenius HIV1/2 in ruling out HIV infection. Conversely, there was a case where both INNO-LIA™ and Multisure HIV1/2 identified a sample as positive, while Geenius HIV1/2 classified it as IND. This suggests that Multisure HIV1/2 may be more effective at accurately recognizing true positive cases compared to Geenius HIV1/2 (Table 1).

The findings of this study highlight the importance of assessing such advanced HIV rapid confirmatory tests, potentially further enhancing and easing diagnostic procedures. Rapid tests have been reliably used as screening tools not only for HIV but also for other infections such as Coronavirus [11,13,15]. Numerous challenges affect current testing strategies, including cost, accessibility, accuracy, and reliability. Advanced rapid tests are promising tools, especially since confirmatory immunoblots face challenges similar to ELISA [13]. Previous studies have reported that immunoblot-based confirmatory assays may present limitations such as false positivity, indeterminate results, and non-specific reactions [9,10,13,15,16,17]. Non-definitive outcomes are strongly undesirable, creating diagnostic uncertainty and requiring extra testing, often causing stress-induced anxiety in patients [16]. Additionally, as mentioned previously, one of the immunoblots’ limitations was its misclassification of HIV-2 [18]. It is seen in a newly conducted study that Geenius HIV1/2 surpasses immunoblots in this regard, where they mention that immunoblot categorized 17% of HIV-2 cases as a dual HIV-1/HIV-2 infection [19]. On the other hand, Geenius HIV1/2 only misclassified 9% of the samples. It has also been suggested that Geenius HIV1/2 was able to outperform immunoblots in sensitivity, where Geenius HIV1/2 sensitivity had a range of 91–100%, while the immunoblot assessed had a sensitivity of 83% [19].

Overall, while both rapid tests demonstrated strong overall performance, a few discrepancies were noted. Each test recorded a single false negative (1/36, 2.8%), and Geenius HIV1/2 had slightly more false positives than Multisure with 6/44 and 4/44, respectively. Additionally, Multisure classified 1/36 PCR-positive samples (2.8%) and 7/44 PCR-negative samples (15.9%) as indeterminate, while Geenius classified 2/36 (5.6%) and 9/44 (20.5%), respectively. Among INNO-LIA™ IND cases (*n* = 27), Multisure resolved 81.5% as negative and only classified 18.5% as IND, whereas Geenius marked 37% as IND. Notably, Multisure misclassified three INNO-LIA™ negatives as IND, while Geenius resolved them correctly. These results highlight the nuanced strengths and limitations of each test, with Multisure HIV1/2 introducing fewer discrepancies overall across false positives, false negatives, and indeterminate classifications.

This study presents several limitations. The relatively small number of indeterminate and discordant samples may affect the generalizability of conclusions drawn from those subsets. The lack of accompanying clinical and serological profiles limited interpretation of unexpected results, especially in the absence of co-infection screening or immune-related context. Although INNO-LIA™ band-level reactivity data were available, comparable band-pattern information from the rapid confirmatory assay (Geenius HIV-1/2) was not provided in an equivalent format. Therefore, direct comparative band-pattern analysis across assays could not be performed and was not included in the study. The analysis was consequently restricted to final interpreted assay outcomes to ensure methodological comparability between platforms. Infection status in this study was determined according to the Medical Commission diagnostic algorithm using reference laboratory assays. However, independent clinical or epidemiological follow-up data, including final resolution of indeterminate cases performed in routine practice, were not available within the study dataset. Therefore, the calculated sensitivity and specificity represent agreement with the reference testing strategy rather than absolute diagnostic accuracy. Although excluding IND results from performance calculations is consistent with standard diagnostic protocols, it limits direct reflection of clinical practice, where such outcomes require interpretation. However, this gap was addressed through a separate IND-focused analysis. In addition, no confirmed HIV-2 infections were present in the analyzed dataset, and HIV-1 subtype information was not available for archived samples; therefore, conclusions regarding HIV-1/2 differentiation rely primarily on assay design rather than clinical HIV-2 case evaluation. Despite these limitations, the study benefited from a comprehensive and diverse sample set, enhancing the robustness of its findings. Moreover, both Multisure and Geenius HIV1/2 demonstrated strong potential as rapid, reliable alternatives to traditional immunoblot assays.

Given that immunoblot-based assays are widely used as confirmatory tests, the emergence of rapid alternatives with comparable performance [46] supports a shift toward more accessible and efficient HIV diagnostics. Within a CDC-aligned diagnostic framework, the Multisure HIV-1/2 assay may function as an alternative antibody differentiation assay at the confirmatory stage following reactive fourth-generation screening, rather than as a secondary test after Geenius.

## 5. Conclusions

In this study, we conducted a comparative evaluation of two rapid HIV-1/2 antibody differentiation assays—Multisure HIV-1/2 and Geenius HIV-1/2—against the reference INNO-LIA™ assay with additional molecular evaluation using PCR. Both rapid assays demonstrated excellent diagnostic performance and high agreement with reference testing strategies. Multisure HIV-1/2 showed a greater ability to resolve indeterminate INNO-LIA™ results, particularly by classifying a higher proportion of such cases as negative. These findings suggest that Multisure HIV-1/2 may represent a practical alternative antibody differentiation assay within CDC-aligned HIV diagnostic workflows, particularly in settings where rapid turnaround, accessibility, and cost considerations are important. However, given the limited number of indeterminate and discordant samples and the absence of confirmed HIV-2 cases, further studies involving larger and more diverse datasets are warranted to validate these findings and to better define the clinical role of this assay.

## Figures and Tables

**Table 1:** A comparative summary of Multisure HIV1/2 and Geenius HIV1/2 assays’ results relative to INNO-LIA™ and PCR.

Test	Multisure HIV1/2			Geenius HIV1/2		
INNO-LIA™	+	−	IND ^‡^	+	−	IND
HIV Positive (*n* ^†^ = 38)	38 (17%)	0 (0%)	0 (0%)	37 (16.5%)	0 (0%)	1 (0.5%)
HIV Negative (*n* = 159)	0 (0%)	156 (69.6%)	3 (1.3%)	0 (0%)	159 (71%)	0 (0%)
HIV IND (*n* = 27)	0 (0%)	22 (9.2%)	5 (2.2%)	2 (0.9%)	15 (6.7%)	10 (4.5%)
Total	224 (100%)			224 (100%)		
**PCR**	+	−	IND	+	−	IND
HIV Positive (*n* = 36)	34 (42.5%)	1 (1.3%)	1 (1.3%)	33 (41.3%)	1 (1.3%)	2 (2.5%)
HIV Negative (*n* = 44)	4 (5%)	33 (41.3%)	7 (8.8%)	6 (7.5%)	29 (36.3%)	9 (11.3%)
Total	80 (100%)			80 (100%)		

^†^ *n*: number of samples; ^‡^ IND: indeterminate; +: Positive; −: Negative.

**Table 2:** A comparative concordance assessment of Multisure HIV1/2 and Geenius HIV1/2 against gold standard INNO-LIA™ and PCR.

Reference	Test *	Sensitivity (%)	Specificity (%)	Positive Predictive Value (%)	Negative Predictive Value (%)	Overall Percent Agreement (%)	Positive Percent Agreement (%)	Negative Percent Agreement (%)	Cohen’s Kappa Coefficient
(CI: 95%)									
INNO-LIA™	Multisure HIV 1/2	100	100	100	100	100	100	100	1.000
		(90.8–100)	(97.7–100)	(90.8–100)	(97.7–100)	(100–100)	(100–100)	(100–100)	(1.000–1.000)
	Geenius HIV 1/2	100	100	100	100	100	100	100	1.000
		(90.6–100)	(97.7–100)	(90.6–100)	(97.7–100)	(100–100)	(100–100)	(100–100)	(1.000–1.000)
PCR ^§^	Multisure HIV 1/2	97.1	89.2	89.5	97.1	93.1	97.1	89.2	0.861
		(85.5–99.9)	(75.3–95.7)	(75.9–95.8)	(85.1–99.9)	(85.8–97.9)	(86.5–99.3)	(74.8–97)	(0.744–0.978)
	Geenius HIV 1/2	97.1	82.9	84.6	96.7	89.9	97.1	82.9	0.797
		(85.1–99.9)	(67.3–91.9)	(70.3–92.8)	(83.3–99.8)	(81.4–97.2)	(86.9–99)	(71.8–94)	(0.657–0.938)

^§^ Polymerase Chain Reaction; * *p* value < 0.001.

## Data Availability

The original contributions presented in this study are included in the article/Supplementary Material. Further inquiries can be directed to the corresponding author.

## Supplementary Materials

The following supporting information can be downloaded at: https://www.mdpi.com/article/10.3390/microorganisms14030693/s1, Supplementary Table S1: Composition of the study sample panel based on Architect HIV Ag/Ab Combo and INNO-LIA™ classification and availability of PCR testing; Supplementary Table S2: Analytical characteristics and antigenic targets of the assays evaluated in the study; Supplementary Table S3: Resolution of INNO-LIA™ indeterminate samples by Multisure HIV-1/2, Geenius HIV-1/2 and HIV-1 PCR.

## Abbreviations

The following abbreviations are used in this manuscript:

Ag/Ab	Antigen/Antibody
AIDS	Acquired Immunodeficiency Syndrome
APHL	Association of Public Health Laboratories
CI	Confidence Interval
CLIA	Chemiluminescent Immunoassay
CDC	U.S. Centers for Disease Control and Prevention
ELISA	Enzyme-Linked Immunosorbent Assay
FDA	Food and Drug Administration
HIV	Human Immunodeficiency Virus
HIV-1	Human Immunodeficiency Virus Type 1
HIV-2	Human Immunodeficiency Virus Type 2
IND	Indeterminate
INNO-LIA™	Line Immunoassay HIV I/II Score
LIA	Line Immunoassay
MC	Medical Commission
MOPH	Ministry of Public Health
NPV	Negative Predictive Value
OPA	Overall Percent Agreement
PCR	Polymerase Chain Reaction
PPA	Positive Percent Agreement
PPV	Positive Predictive Value
RDT	Rapid Diagnostic Test
WHO	World Health Organization
