# Supplementary material for: Advancing HIV Diagnostics: Comparative Evaluation of Multisure HIV-1/2 Rapid Confirmatory Test Against Geenius and Traditional Reference Assays Within a CDC-Aligned Diagnostic Framework
Source: Microorganisms. 2026 Mar 19;14(3):693. doi: 10.3390/microorganisms14030693 (PMC13028974; doi:10.3390/microorganisms14030693)
Supplement: Supplementary file 1 [file microorganisms-14-00693-s001.zip › microorganisms-4115052-supplementary.pdf]

Supplementary Table S1: Composition of the study sample panel based on Architect HIV Ag/Ab Combo and INNO-LIA™ classification and availability of PCR testing.

Group	Definition	n	PCR Performed
Concordant positive	Architect reactive / INNO-LIA™ positive	38	Yes
Concordant negative	Architect non-reactive / INNO-LIA™ negative	139	No
Discordant	Architect reactive / INNO-LIA™ negative	20	Yes
Indeterminate (PI)	Architect reactive / INNO-LIA™ indeterminate	22	Yes
Indeterminate (NI)	Architect non-reactive / INNO-LIA™ indeterminate	5	No
**Total = 224**			

Supplementary Table S2: Analytical characteristics and antigenic targets of the assays evaluated in the study

Assay	Method type	Target detected	Differentiates HIV-1/2	Main antigens/markers detected
Architect HIV Ag/Ab Combo	4th-gen Ag/Ab CMIA	p24 antigen + antibodies	No	Antibodies to HIV-1 (groups M & O) and HIV-2 + HIV-1 p24 antigen
INNO-LIA™ HIV I/II Score	Line immunoassay (recombinant immunoblot)	Antibodies	Yes	Recombinant HIV-1 (sgp120, gp41, p31, p24, p17) & HIV-2 (gp36, gp105)
Geenius HIV-1/2 Supplemental	Rapid antibody differentiation immunoassay	Antibodies	Yes	HIV-1 (gp160, gp41, p24) & HIV-2 (gp36, gp140)
Multisure HIV-1/2 Confirmatory	Rapid antibody immunochromatographic assay	Antibodies	Yes	HIV-1 (gp120, gp41, p24) & HIV-2 (gp36, gp105)
HIV-1 PCR	Nucleic acid amplification	Viral RNA	No	HIV-1 RNA

Supplementary Table S3: Resolution of INNO-LIA™ indeterminate samples by Multisure HIV-1/2, Geenius HIV-1/2 and HIV-1 PCR

**INNO-LIA indeterminate subgroup**		Architect reactive (PI)	Architect non-reactive (NI)	**Total**
**n**		22	5	**27**
**Multisure**	**Positive**	0	0	0
	**Negative**	17	5	22
	**IND**	4	0	4
**Geenius**	**Positive**	2	0	2
	**Negative**	10	5	15
	**IND**	9	0	9
**PCR ***	**Positive**	2	0	2
	**Negative**	20	0	20
	**Not performed**	0	5	5

*PCR testing was performed only for Architect-reactive samples according to the diagnostic workflow
